# Functional Tic-like Behaviors: From the COVID-19 Pandemic to the Post-Pandemic Era

**DOI:** 10.3390/healthcare12111106

**Published:** 2024-05-28

**Authors:** Andrea Eugenio Cavanna, Laura Spini, Silvia Ferrari, Giulia Purpura, Anna Riva, Renata Nacinovich, Stefano Seri

**Affiliations:** 1 Department of Neuropsychiatry, National Centre for Mental Health, BSMHFT and University of Birmingham, Birmingham B15 2FG, UK; 2 School of Life and Health Sciences, Aston Brain Centre, Aston University, Birmingham B4 7ET, UK; 3 Sobell Department of Motor Neuroscience and Movement Disorders, Institute of Neurology, University College London, London WC1E 6BT, UK; 4 Department of Child Neuropsychiatry, IRCCS San Gerardo dei Tintori, 20900 Monza, Italy; 5 School of Medicine and Surgery, University of Milano-Bicocca, 20125 Milan, Italy

**Keywords:** functional tic-like behaviors, neurodevelopmental tics, tic-like behaviors, Tourette syndrome

## Abstract

During the COVID-19 pandemic, there have been multiple reports about an unforeseen surge in adolescents and young adults exhibiting sudden onset functional tic-like behaviors. This phenomenon has been mainly associated with the female gender and occasionally after exposure to social media content featuring similar patterns of functional tic-like behaviors. A significant portion of these individuals have been directed to specialist clinics for movement disorders with initial misdiagnoses of late-onset refractory Tourette syndrome. Distinguishing between rapid onset functional tic-like behaviors and neurodevelopmental tics as part of Tourette syndrome can be challenging; however, the differential diagnosis is facilitated by focusing on specific clinical and demographic factors, which we have explored in a systematic literature review. Compared to neurodevelopmental tics, functional tic-like behaviors typically present with a more abrupt and intense manifestation of symptoms, onset at a later age, higher prevalence among females, inability to suppress tics, coexisting anxiety and depression, and sometimes a history of exposure to social media content portraying tic-like behaviors of a similar nature. This novel manifestation of a functional neurological disorder may thus be viewed as an emerging neuropsychiatric condition potentially triggered/exacerbated by the psychosocial repercussions of the COVID-19 crisis.

## 1. Introduction

Functional tic-like behaviors are a subtype of functional movement disorder consisting of movements or vocalizations that resemble neurodevelopmental tics [1,2,3,4,5,6]. According to the scientific literature published before the COVID-19 pandemic, functional tic-like behaviors were a relatively rare manifestation of functional movement disorder [7,8,9,10,11,12,13,14,15,16,17]. This perception has changed since the beginning of the pandemic because of an unprecedented increase in patients with functional tic-like behaviors seen in movement disorder clinics worldwide [1,2,3,4,5,18]. Movement disorders specialists in the United States reported a 60% increase (90% in pediatric age, 51% in adult age) in new patients diagnosed with functional movement disorders during the first 8 months of the COVID-19 pandemic. Of note, 4/45 (9%) patients newly diagnosed with functional movement disorders had functional tic-like behaviors [19]. A similar trend was reported at a specialist movement disorders clinic in France, where functional movement disorders represented 9% of all patients hospitalized for movement disorders during the first 15 months of the COVID-19 pandemic—versus the usual 4% observed before COVID-19. This increase was referred to as an additional ‘silent epidemic’ alongside the COVID-19 pandemic, and paroxysmal hyperkinetic movements accounted for 10/39 (26%) of emergency referrals for functional movement disorders [20]. A population-based study carried out in England documented a more than fourfold increase in teenage girls who developed tic-like behaviors during the COVID-19 pandemic, often in association with anxiety and/or other mental health co-morbidities [21].

Functional tic-like behaviors reported in recent years primarily occur in adolescents or young adults, and females appear to be at higher risk. These demographic features, together with the absence of a family history of tics in most cases, differentiate patients with functional tic-like behaviors from those with neurodevelopmental tic disorders [1,2,3,4,5,18]. Moreover, functional tic-like behaviors are characterized by a (sub)acute onset and often manifest a disproportionately higher prevalence of complex vocalizations and repetitive movements involving the limbs. These clinical features differ substantially from the phenomenology and patterns that are typical of neurodevelopmental tics [1,2,3,4,5,18]. The clinical phenotypes of patients with onset of functional tic-like behaviors during the pandemic have been documented in reports from the United States [22,23], the United Kingdom [24,25,26], and multiple European countries [27,28,29], as well as a multi-national registry collating data from ten specialist centers across North America, Australia, and Europe [30]. Moreover, phenotypical differences between patients with functional tic-like behaviors and patients with neurodevelopmental tics have been explored in multiple controlled studies from the same three continents [31,32,33,34,35,36,37,38,39,40,41,42].

Patients with functional tic-like behaviors often present with co-occurring anxiety or affective symptoms and can report significant psychosocial stressors. The psychological impact of the COVID-19 pandemic and lifestyle changes caused by the pandemic might have played a role in the concomitant increase in functional tic-like behaviors [18]. Interestingly, a substantial proportion of patients with functional tic-like behaviors exhibit characteristics mirroring alleged representations of tics in social media they were exposed to during the pandemic. Expert assessments of popular videos claiming to portray Tourette syndrome on platforms like TikTok indicated that such portrayals were largely inaccurate or atypical [43,44]. Specifically, Tourette syndrome portrayals in these videos predominantly focused on environmentally triggered complex behaviors, including aggressive actions, self-harming behaviors, throwing objects, and coprolalic vocalizations. This strongly indicates that functional tic-like behaviors developing after social media consumption differ significantly from neurodevelopmental tics observed in Tourette syndrome [31]. The term ‘mass social media-induced illness’ was proposed to refer to this outbreak of a new type of mass sociogenic illness that, in contrast to all previously reported episodes, is spread solely via social media [45].

The present review aims to provide the state of the art on the newly described phenomenon of functional tic-like behaviors developed during the COVID-19 pandemic, with a focus on their clinical phenomenology and its implications for the diagnosis and treatment of this patient population.

## 2. Materials and Methods

### 2.1. Systematic Literature Search

We conducted a systematic literature review according to the guidelines described in the Preferred Reporting Items for Systematic Reviews and Meta-Analyses (PRISMA) 2020 statement [46]. Comprehensive systematic searches of the PubMed, Embase, and PsycInfo databases were completed for this review. As for the search strategy, we restricted the searches to studies focusing on functional tic-like behaviors by using the following search terms: “functional tics” OR “functional tic-like behaviors” OR “psychogenic tics”. Once the searches were completed, titles and abstracts were screened according to eligibility criteria. If a decision for eligibility was not able to be made at the title and abstract screening stage due to insufficient information, the full article was reviewed. Following this, the full texts of identified studies were further screened with reasons for exclusion noted. Reference lists of studies were hand-searched to check if any potential studies were not captured by the search strategy. A data extraction template was designed to include a descriptive summary of the studies included in the review [47]. Extracted data for the included studies were as follows: authors, year, country, setting, size of the clinical sample (with age and gender distribution), clinical characteristics, and predictors/correlates of functional tic-like behaviors, as well as other study findings in relation to the review question. Three researchers (L.S., S.F., and A.E.C.) were involved in the data extraction process, and any discrepancies were resolved through open discussion between the researchers.

### 2.2. Eligibility Criteria

Studies were included in our systematic literature review if they met pre-defined eligibility criteria regarding participants. We included studies of functional tic-like behaviors developed by patients of all ages since the outbreak of the COVID-19 pandemic (no restrictions on time since the onset of the pandemic in 2020). We excluded studies focusing on tics due to any neurological disorder (secondary tics). As for publication type and study design, we included in our systematic literature review original quantitative studies conducted according to cross-sectional, observational, cohort, and case-control protocols. We did not set any limits in terms of context. Studies across different settings such as specialist clinics and independent living in the community were included. We excluded qualitative studies, reviews, and unpublished ‘grey’ literature. Studies published in languages other than English were also excluded.

## 3. Results

**Figure 1:**
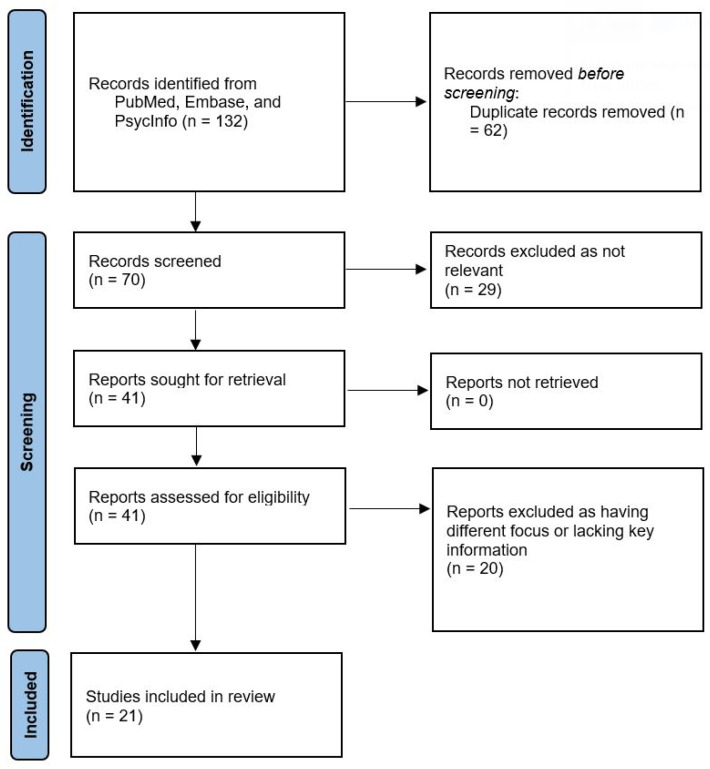
PRISMA flow diagram illustrating the study selection process.

Initial searches of the scientific databases yielded 132 results, with a total of 70 articles once duplicates were removed. The titles and abstracts were assessed for eligibility and articles were excluded if they did not meet the review criteria (29 articles). A total of 41 articles were reviewed in full-text and reasons for exclusion at this stage were recorded. Following eligibility checking, 21 studies were regarded as eligible for the review: 9 case series and 12 controlled studies of new-onset functional tic-like behaviors during the COVID-19 pandemic. The PRISMA flowchart displaying the selection process of the reviewed studies is shown in Figure 1.

We retrieved nine case series of new-onset functional tic-like behaviors during the COVID-19 pandemic. A summary of the reviewed reports is provided in Table 1.

The reports were published between 2021 and 2023. Three case series were from the United Kingdom [24,25,26], two from the United States [22,23], and one each from Denmark [29], Germany [27], and Hungary [28]; the largest series came from an international registry that collated data from ten specialist centers across North America, Australia, and Europe [30]. All the reported cases were seen at specialist clinics for patients with tic disorders, except for one small cluster of high school students experiencing functional tic-like behaviors [23]. These reports were characterized by a wide range of sample sizes, ranging from 5 to 294 patients. The mean ages of the patients were between 12 and 19 years. The majority of patients were females across all studies (proportions of females ranging from 72% to 100%), with the exception of the study conducted in Germany, where half of the patients were males [27].

Additionally, we retrieved 12 original studies providing comparative data between the characteristics of functional tic-like behaviors with onset during the COVID-19 pandemic and the phenomenological features of neurodevelopmental tics. A summary of the reviewed studies is provided in Table 2.

The timeframe of the studies spans four years (between 2021 and 2024), and the geographic distribution is skewed towards North America, where the majority of the studies took place. Specifically, five studies were conducted in Canada [32,33,39,41,42], two each in Germany [31,40] and in the United States [35,37], and one each in Australia [34], Denmark [36], and the United Kingdom [38]. All studies were conducted within specialist clinic settings. Sample sizes ranged between 9 and 83 patients. The mean ages of the patients with functional tic-like behaviors were between 14 and 32 years. At least half of the patients were females across all studies (proportions ranging from 50% to 100%), except for one study conducted in the United States [37] and one study conducted in Germany [31], where 48% and 38% of the patients were females, respectively. The comparison groups of patients with neurodevelopmental tics were characterized by a lower proportion of female patients, except for two studies where matched controls were recruited [31,38].

## 4. Discussion

Possible descriptions of functional tic-like behaviors have been reported as early as 1884, pre-dating Georges Gilles de la Tourette’s original description of the condition, characterized by multiple neurodevelopmental tics that bear his name, by one year [48]. However, most of what is currently known about functional tic-like behaviors has been learned during the COVID-19 ‘pandemic within the pandemic’ [43]. It has been pointed out that the scattered reports of functional tic-like behaviors before the pandemic [7,8,9,10,11,12,13,14,15,16,17] have been inconsistent, due to relatively small sample sizes, selection bias of the populations studied, and a lack of standardized diagnostic criteria [18].

The picture changed since the onset of the COVID-19 pandemic. Three early reports described five [28], six [22], and ten female patients [25] with acute-onset functional tic-like behaviors, respectively. Despite the low number of patients involved, these initial case series alerted the scientific community about a new clinical phenomenon detected in both North America and Europe, concomitant with the ongoing pandemic. Of note, two of these small series (average age: 14 years) flagged a possible association with social media consumption [22,28], whereas the third one focused on younger patients (12 years old) and included practical advice for the management of functional tic-like behaviors in an educational setting [25]. Exposure to tic-like behavior on social media before symptom onset was reported by 27 out of 28 adolescents (all females but one) from a Danish specialist clinic [29]. Two larger case series of 34 adolescents [24] and 32 young adults [27] highlighted the co-morbidity burden, including co-existing neurodevelopmental tics in approximately half of the patients. The largest series of functional tike-like behaviors from a single center reported data on 105 adolescents and young adults [26]. Co-morbid anxiety and affective disorders were prevalent, and previous exposure to tic-like behavior on social media was reported by half of the sample. The most reliable data originated from an international registry involving Canada, the United Kingdom, Germany, Australia, the United States, Italy, France, and Hungary [30]. Collated data (*n* = 294) confirmed previous observations of acute onset and female gender preponderance. The patients were seen at ten tertiary referral centers for tic disorders over two and half years during the COVID-19 pandemic and were more likely to present with anxiety and depression than with other neurodevelopmental disorders in co-morbidity. Moreover, almost 60% of patients explicitly reported exposure to tic-related social media content. Apart from the small case series by Firestone et al. [23], which described eight female students from the same high school, all the patients reported in the case series were seen in specialist settings and were therefore subject to possible referral bias. Interestingly, all the students from the school cluster had a history of anxiety and/or affective symptoms and reported having been exposed to tic behaviors in person (*n* = 8) and through social media (*n* = 3) [23].

The first controlled study comparing the clinical features of functional tic-like behaviors with those of neurodevelopmental tics was conducted at a Canadian specialist clinic on nine female adolescents and young adults who developed their symptoms after exposure to tic-like behavior on social media [33]. The authors recruited 24 controls from their tic registry [33] and were able to document significantly older age at onset and more complex clinical phenomenology in patients with functional tic-like behaviors. These findings were replicated in a larger study from the same group, where 20 adolescents (all females but one) with functional tic-like behaviors developed after exposure to tic-like behavior on social media were compared to 270 patients with neurodevelopmental tic disorders from the local registry [32]. An early controlled study from Germany, despite the small sample size (*n* = 13), yielded at least two interesting findings [31]. First, most patients (8/13) were males: as noted by the authors, all patients developed functional tic-like behaviors after social media exposure—in Germany, a popular social medial content developer is a young male “allegedly featuring ‘Tourette syndrome’, but in fact showing complex behavior, elaborated swearing, and offensive phrases” [31]. Second, a comparison of the clinical phenomenology between the index cases and matched patients with neurodevelopmental tics previously assessed at the same center confirmed the presence of more complex symptomatology in the functional group. Later age of onset, higher symptoms complexity, and higher rates of co-morbid anxiety and depression were also reported in a controlled study of 22 female adolescents from an Australian specialist clinic [34]. In this study, control data were retrospectively collected from 163 patients with neurodevelopmental tics seen at the same center.

Trau et al. [35] assessed a clinical sample of children and adolescents with multiple tic types to create a patient-based diagnostic checklist for the differential diagnosis between patients with functional tic-like behaviors (*n* = 36), patients with neurodevelopmental tics (*n* = 119), and patients with a previous history of neurodevelopmental tics who acutely developed a functional overlay (*n* = 25). Of note, in this study, age at tic onset was not found to be significantly different between the groups. Conversely, in a study conducted in Denmark, 53 youths (of whom 50 were females) were older at symptom onset than 200 controls with neurodevelopmental tics [36]. Interestingly, they were also more likely to have experienced an adverse psychosocial event prior to symptom onset. Comparative data for young adults with functional tic-like behaviors were obtained from two clinical databases in the United States (*n* = 21) [37] and in the United Kingdom (*n* = 83) [38]. In the latter study, patients with functional tic-like behaviors and controls with neurodevelopmental tics were matched for age and gender. In a similar study conducted on a German sample (*n* = 32), half of the patients with functional tic-like behaviors were males and were not more likely to present with co-morbid anxiety and depression than patients with neurodevelopmental tics [40]. Finally, three studies were recently conducted at the same specialist clinic in Canada on 35 [39], 40 [42], and 41 [41] adolescents and young adults with functional tic-like behaviors, respectively. In addition to confirming a significantly higher proportion of females with older age at symptom onset, these studies found that patients with functional tic-like behaviors were more likely to present with anxiety and depression [39], to be gender-diverse individuals [42], and to report coprophenomena, popping, whistling, simple head movements, and self-injurious behaviors [41] than patients with neurodevelopmental tics. Since all controlled studies were conducted in specialist clinics, referral bias needs to be taken into account when interpreting their findings, with regard to both cases with functional tic-like behaviors and controls with neurodevelopmental tics.

The quality of the available evidence supporting the use of selected clinical features to accurately differentiate patients with functional tic-like behaviors from patients with Tourette syndrome is variable. Based on the reviewed studies, certain features (from patients’ demographics to tic phenomenology) appear to be more useful than others in diagnosing individual patients. Crucially, clinicians are not supposed to rely on a single feature to validate their diagnosis, and it is often helpful to evaluate the evolution of the clinical presentation over time and exercise diagnostic humility pending further information [49].

Differences in demographic and clinical characteristics have been observed since the first reports of tic-like behaviors in the early stages of the COVID-19 pandemic. Neurodevelopmental tics are 3–4 times more common in males than females, and patients with Tourette syndrome and other neurodevelopmental tic disorders develop their initial symptoms around the age of 5–6 years [50,51]. A striking female preponderance was noted across all clinical samples of patients with functional tic-like behaviors, except for one sample from the United States [37] and the reports from Germany [27,31,40]. Of note, the clinical presentation of the patients from Germany closely resembled the jerky movements and complex utterances portrayed by a popular male influencer who achieved high popularity in that country. The proportion of females was significantly higher in patients with functional tic-like behaviors than in those with neurodevelopmental tics across all unmatched controlled studies. As for the age at symptom onset, functional tic-like behaviors were first reported in adolescence and early adulthood across all the reviewed studies, at a significantly later developmental stage compared to Tourette syndrome [50,51]. A family history of neurodevelopmental tics can be elicited more frequently in patients with Tourette syndrome than in patients with functional tic-like behaviors [36,38]. The neuropsychiatric spectrum of Tourette syndrome has been consistently characterized as encompassing other neurodevelopmental conditions, especially attention-deficit and hyperactivity disorder [50,51]. Moreover, tic-related obsessive-compulsive symptoms of different degrees of severity can be reported by the majority of patients with neurodevelopmental tics but are rarely seen in association with functional tic-like behaviors [52]. Patients with Tourette syndrome are often diagnosed with co-morbid anxiety and affective disorders [53,54]; however, these symptoms seem to present with a higher prevalence in patients with functional tic-like behaviors [23,24,25,26,39].

An accurate evaluation of the time course of tic evolution can provide valuable diagnostic clues. Neurodevelopmental tics are characterized by a gradual onset, with spreading and worsening over the course of years, whereas functional tic-like behaviors have typically been associated with a (sub)acute onset [23,27,30,38]. It has also been reported that functional tic-like behaviors do not usually present with the rostro-caudal distribution or progression that is characteristic of neurodevelopmental tics. Case series providing accurate clinical descriptions of functional tic-like behaviors and studies comparing their phenomenology with that of neurodevelopmental tics provided initial data suggesting differences in the characteristics of the tic repertoire. The phenomenological features of functional tic-like behaviors tend to involve specific complex movements/actions, often including (self) hitting [27,32,35,36,38], throwing objects [25,27,32,38], or freezing/motor blocking [35,38,41]. This contrasts with the higher prevalence of simple motor tics in patients with Tourette syndrome and other neurodevelopmental tic disorders, especially in the early stages of the condition [55,56]. Among the motor manifestations, the most reliable indicator of functional tic-like behaviors versus neurodevelopmental tics seems to be the higher prevalence of self-injurious tics, such as chest or head banging [57]. Patients with functional tic-like behaviors often present with complex vocal tics that include bizarre context-dependent words and statements, as well as coprolalia [27,30,33,35,36,38,40,41], which are reported by a minority (10–30%) of patients with Tourette syndrome or other neurodevelopmental tic disorders [58,59]. Conversely, a higher prevalence of simple vocal (phonic) tics, especially throat clearing, has been shown to be significantly more suggestive of neurodevelopmental tics [57]. Preliminary evidence indicates that a high prevalence of complex vocal tics might be a stronger indicator of functional tic-like behaviors than a high prevalence of complex motor tics. The results of a recent cross-sectional study suggested that simple head movements (e.g., neck jerking) and complex vocalizations (e.g., enunciation of words, including swear words) were the movements and vocalizations that were most strongly associated with functional tic-like behaviors in comparison to neurodevelopmental tics [57].

The diagnostic validity of functional tic-like behaviors as distinct from neurodevelopmental tics was recently confirmed by a study showing that the presenting symptoms of functional tic-like behaviors differ substantially from new-onset neurodevelopmental tics in patients who are later diagnosed with Tourette syndrome [60]. Six clinical features were listed as having a positive predictive value over 90% for functional tic diagnosis if the prior probability is 50%. These were movements or vocalizations that are dramatically worse in the presence of others versus when alone, coprophenomena at onset, coprolalia at presentation, symptoms that dramatically and persistently disrupt the person’s intended actions or communications, ‘tic attacks’, and severe symptoms at onset [60]. Other features, such as the presence of subjective sensory phenomena or premonitory urges to tic, seem to be characterized by a remarkably low specificity, as they are commonly reported in association with both functional and neurodevelopmental tics [61,62].

It is important to reiterate that although certain features are significantly more common in patients diagnosed with functional tic-like behaviors, their presence does not automatically rule out a diagnosis of neurodevelopmental tics. For example, complex behaviors can sometimes be part of the neurodevelopmental tic repertoire of patients with Tourette syndrome, and conversely, patients with functional tic-like behaviors can sometimes present with a preponderance of simple tics. The phenomenological overlap between neurodevelopmental tics and functional tic-like behaviors poses significant challenges to the differential diagnostic process, highlighting the need for a more in-depth and comprehensive evaluation that takes into account other clinical characteristics, as well as demographic variables. An analysis conducted recently through video observation by field experts on functional tic-like behaviors and/or neurodevelopmental tics revealed the challenge of distinguishing between the two solely based on the observed movement disorder [63]. Clinicians require additional information regarding clinical history such as onset, symptom evolution, and the presence of contextual triggers. Nevertheless, the authors of this study emphasized that phenomenology was deemed the pivotal factor in diagnostic recognition [63]. Finally, it has been suggested that, in the absence of established diagnostic criteria consistently used across studies, the diagnostic process and clinical characterization of patients with functional tic-like behaviors may suffer from a degree of circularity [49]. For example, if clinicians expected to see more complex behaviors in patients with functional tic-like behaviors and based their diagnostic approach on the higher prevalence of complex behaviors, they would potentially classify a higher number of patients with complex behaviors in the functional tic group.

## 5. Diagnostic Checklists for Functional Tic-Like Behaviors

The European Society for the Study of Tourette Syndrome (ESSTS) published expert consensus on diagnostic criteria to support the differential diagnosis between patients with functional tic-like behaviors and patients with neurodevelopmental tics, based on three major and two minor diagnostic criteria [64]. The three major diagnostic criteria are (1) age at first symptom onset of 12 years or older, (2) rapid onset and evolution of symptoms (patient and family can often pinpoint the date/circumstances of symptom onset and clinical presentation evolves over hours/days, typically increasing to peak severity over a period of a few weeks/months), and (3) the presence of at least four out of nine phenomenological features (multiple tic-like movements and/or vocalizations occur, with a larger number of complex than simple tic-like behaviors; the same tic-like behavior has an inconsistent rather than stereotyped presentation; motor tic-like behaviors include complex arm and hand movements such as banging chest/head, tapping, hitting others, sign language, throwing objects, offensive gestures, drop attacks, or freezing, and can be context-dependent, or violent and ballistic, potentially leading to self-injury or damage to objects; motor tic-like behaviors do not to follow the typical rostro-caudal progression in their first appearance; vocal tic-like behaviors include context-dependent and offensive words/statements; tic-like behaviors resemble popular cultural influences/references or individuals in the patient’s social environment; patients experience a large variation in symptom frequency and intensity over the course of a single day, with symptom-free activities for several hours, and severe symptoms in specific contexts; tic-like behaviors change rapidly, with the constant onset of new tic-like behaviors on a daily basis or every few days; the examining clinician observes an increase in tic-like behaviors during the physical examination of the patient). Minor diagnostic criteria are (1) specific comorbidity profile with predominant anxiety/depression and (2) the presence of other functional neurological symptoms. The ESSTS panel proposed that a ‘clinically definite’ diagnosis of functional tic-like behaviors requires the presence of all three major criteria, whereas a ‘clinically probable’ diagnosis of functional tic-like behaviors requires the presence of two major criteria plus one minor criterion. The specificity of individual ESSTS criteria for the diagnosis of functional tic-like behaviors (age at onset and selected features of the phenomenological criterion) was recently tested in a large sample of youth with Tourette syndrome at the time of first visit to a specialist clinic [57]. The high specificity of the age at onset criterion and having at least one complex vocal and two complex motor tics at the time of the first visit were observed. A few of the complex motor tics had lower specificity (e.g., complex arm and hand movements and self-injurious behaviors) when assessed individually. Based on their findings, the authors concluded that the requirement in the ESSTS expert consensus to fulfill at least four out of nine phenomenological criteria for the diagnosis of functional tic-like behaviors appears justified.

A group based in the United States also proposed a set of three working criteria as part of a diagnostic checklist for functional tic-like behaviors during the COVID-19 pandemic [35]. According to these criteria, functional tic disorder (1) must be sudden and fulminant in onset, (2) must include at least one out of three patient characteristics (co-morbid anxiety, female sex, lack of family history), and (3) must have at least two out of seven tic features (tic attacks, blocking tics, frequent coprolalia or coprophenomenon, a broad spectrum of word use or frequent coprolalia, self-injurious tics or tics injurious to others, throwing, and the presence of other functional neurological symptoms). Of note, the age at tic onset criterion is missing from these criteria, which include the female gender criterion (absent in the ESSTS guidelines).

## 6. Treatment of Functional Tic-Like Behaviors

The level of impairment caused by functional tic-like behaviors was assessed in a retrospective, cross-sectional study conducted in a pediatric movement disorders clinic in the United States [65]. A total of 89 youths newly diagnosed with functional tic-like behaviors were compared with 89 youths newly presenting for evaluation of Tourette syndrome. The two groups of patients completed the Mini-Child Tourette Syndrome Impairment Scale (mini-CTIM), a validated clinical tool for assessing tic- and non-tic-related impairment in home, school, and social settings, rated by children and adolescents with tic disorders (mini-CTIM-C) and by their parents (mini-CTIM-P) [66,67]. Impairment ratings regarding home, school, and social environments as assessed by the mini-CTIM were similar for functional tic-like behaviors and neurodevelopmental tics. However, functional tic-like behaviors were more commonly associated with reported emergency department visits, physical injury, and homeschooling, with likely detrimental impacts on individual and family function, as well as health-related quality of life. A recent investigation into mothers’ experiences of their children’s functional tic-like behaviors and their attempts to access support services in the United Kingdom shed further light on the impact of this condition [68]. Thematic analysis of semi-structured interviews with 21 mothers of young people with functional tic-like behaviors (age range 12–17 years) revealed gaps and inconsistencies within the process of gaining access to professional services and a lack of support for the management of functional tic-like behaviors, in addition to highlighting their impact on daily family life. The generated themes encompassed the onset and progression of functional tic-like behaviors, the severity and duration of symptoms, the emotional toll on the family, and the necessity for establishing a well-defined care plan. Coping with functional tic-like behaviors and concurrent issues such as suicidal thoughts and self-harm, alongside the physical and emotional strain, often led to feelings of isolation and helplessness, hindering the family’s functioning and societal participation. These findings underscore the pressing requirement for a structured approach to managing functional tic-like behaviors, including the availability of knowledgeable professionals, enhanced communication with families during referrals, and a focus on addressing anxiety and other mental health issues identified.

Some interventions for neurodevelopmental tics show limited efficacy for functional tic-like behaviors. Specifically, pharmacotherapy for neurodevelopmental tics is not effective in functional tic-like behaviors, whereas there is some evidence that behavioral therapy used for the treatment of neurodevelopmental tics could also be effective in functional tic-like behaviors [18]. However, evidence-based management strategies for patients who developed functional tic-like behaviors during the pandemic are lacking. The treatment approach for patients with functional tic-like behaviors should be tailored to the individual patient. An effective treatment plan should involve educating the patient and his family about the nature of functional tic-like behaviors, avoiding unnecessary interventions and treatments, as well as identifying and mitigating triggers and exacerbating factors. Specifically, psychoeducation can foster the necessary acceptance of the diagnosis, which is an important predictor of a favorable prognosis, alongside the effective management of psychological stressors and psychiatric co-morbidities [18]. Cognitive-behavioral therapy (including remote and self-guided delivery) has been suggested as beneficial, based on its proven efficacy and safety in patients with functional neurological disorders, as well as its established use for the treatment of concurrent anxiety, affective symptoms, and sleep problems [18]. Serotonergic pharmacotherapy has been used with good results for the treatment of psychiatric co-morbidities [18]. Expert advice also highlights that it is crucial to carefully avoid behaviors that reinforce the symptoms [49]. Targeted interventions aimed at reducing both the occurrence of functional tic-like behaviors and their reinforcement should be tested and validated through research trials. To date, only two studies on treatment interventions specifically developed for this patient population have been published: a cognitive behavioral intervention [69] and a psychoeducation group [70] for youths with functional tic-like behaviors. A group based in Australia implemented the Integrated Cognitive Behavioral Intervention for Functional Tics (I-CBiT), incorporating an urge acceptance model originally developed to address neurodevelopmental tics and associated stress and anxiety [69]. The authors of this study enrolled eight young individuals with new and sudden onset functional tic-like behaviors who underwent I-CBiT, combining traditional behavioral tic interventions with third-wave cognitive behavioral therapies in a highly personalized strategy. The intervention, consisting of psychoeducation, exposure and response prevention with urge acceptance, sensory grounding strategies, and cognitive behavioral techniques targeting stress arousal, was completed in three phases. Before and after treatment, functional tic-like behavior severity and impairment were assessed, revealing a significant reduction in functional tic-like behavior severity and improvement in daily functioning for all participants. These cases underscored the importance of urge acceptance in managing functional tic-like behaviors, emphasizing the necessity of addressing underlying stress and anxiety for lasting change. The implementation and evaluation of an online psychoeducation group for youth experiencing functional tic-like behaviors was recently published by a group based in the United Kingdom [70]. Across six groups run by clinical psychologists, 50 participants completed pre- and post-group goal-based assessments, with 36 participants providing service-user feedback incorporating both qualitative and quantitative data about their experiences. Both young participants and their parents reported significant improvements in goal-based outcomes following the group sessions, along with increased knowledge and confidence in managing functional tic-like behaviors. These findings highlighted the acceptability and effectiveness of a virtual psychoeducation group intervention as an initial step in treating functional tic-like behaviors in young individuals.

## 7. Functional Tic-Like Behaviors in the Post-Pandemic Era

With few exceptions [71], it has been shown that the COVID-19 pandemic and the pandemic-related changes have had a considerable impact on patients with neurodevelopmental tics [72,73,74,75,76]. As with the general population [77], complex long-term effects of this global phenomenon have been documented in patients with Tourette syndrome [78]. Further longitudinal studies are needed to fully understand the long-term trajectories of functional tic-like behaviors in the post-pandemic era. As we transition to the post-pandemic era, relatively little is known about the long-term prognosis of patients with functional tic-like behaviors. Preliminary data suggested an overall benign outcome. A prospective cohort study [79] described the clinical course of 29 patients (20 adolescents and 9 adults) with rapid-onset functional tic-like behaviors, previously reported in two case series from Canada [32,33]. Tic severity ratings were significantly improved at 6-month follow-up, with evidence of response to management of co-morbid anxiety and affective symptoms with serotonergic agents and cognitive behavioral therapy, especially in the adolescent group. Follow-up data were presented for a cohort of 32 patients diagnosed with mass social media-induced illness following exposure to a social media influencer portraying tic-like behaviors in Germany [27]. Over half (53%) of the patients reported a significant improvement (average improvement of 74%) in their functional tic-like behaviors after a mean follow-up period of 5 months. In a case series from a specialist clinic for children and adolescents with tic disorders in Italy, the clinical course of 11 patients with acute-onset functional tic-like behaviors (mean age 15 years) was prospectively evaluated with serial follow-up assessments [80]. At the 12-month follow-up, patients with functional tic-like behaviors reported a significant improvement in the severity of functional tic-like behaviors and anxiety symptoms, despite persistent obsessive-compulsive behaviors and affective symptoms. A review of electronic medical records from a clinic based in the United States provided further data on the prognosis of pediatric patients with functional tic-like behaviors during the pandemic. As many as 21 out of 29 patients (82%) reported at least some improvement at a median follow-up time of over 6 months after diagnosis [81]. Analysis of possible predictors of clinical trajectories revealed greater age and longer time to diagnosis as factors decreasing odds of improvement within one month of diagnosis.

To date, the most informative data on the prognosis of functional tic-like behaviors have been provided by two studies from North America, one retrospective study [82] and one prospective study [83] conducted on larger samples and over longer follow-up times. A retrospective follow-up study in the post-COVID-19 isolation era was conducted on 56 patients (age range 10–18 years) at Boston Children’s Hospital, United States [82]. Most patients (96%) were female-assigned at birth and 45% were gender-diverse. The average duration of follow-up was 17 months. Up to 79% of patients improved independently of the co-morbidity pattern or treatment intervention. A subset of patients reported improvement in their functional tic-like behaviors, but not in their general functioning, and continued to present with other functional symptoms. Of note, behavioral interventions developed for the treatment of neurodevelopmental tics were not found to be more effective than mental health therapy alone. The authors of this study remarked that such a trend towards improvement independent of treatment highlights the unique pathophysiology of functional tic-like behaviors. A total of 83 youths and adults with functional tic-like behaviors from clinical tic disorder registries at the University of Calgary, Canada, were prospectively evaluated to characterize the trajectory of symptom severity over a 12-month period after their first clinical visit [83]. Tic severity ratings decreased significantly from the first clinical visit to 6 months (58 patients) and from 6 to 12 months (32 patients). Tic severity at initial presentation and the presence of other functional neurological symptoms were found to be associated with higher tic severity at 6 months, whereas younger age at baseline, serotonergic pharmacotherapy, and cognitive behavioral therapy for co-morbid anxiety and affective symptoms were associated with lower tic severity at 6 months.

## 8. Open Questions

Several open questions remain, translating into specific areas for further study. Although the phenomenology of functional tic-like behaviors has been described in many cohorts and studies since the beginning of the pandemic, there is a need to establish the accuracy of clinical clues to support the diagnostic process. It would be important to conduct further investigations measuring the consistency of reported phenomenological features across different centers, with an exploration of the possible reasons for discrepancies. The possible role of a popular male influencer in determining the unique gender ratio of patients with functional tic-like behaviors in Germany [45] is but one example of this interdisciplinary field of investigation. Overall, the effects of age at onset and gender on functional tic-like behaviors, as well as the influence of reinforcing environmental responses, deserve further investigation. The search for endophenotypes could shed light on the pathophysiology of functional tic-like behaviors and its possible overlap with what is known about different presentations of functional neurological disorders [84].

Interestingly, there is preliminary evidence that in some patients, pre-existing tics and Tourette syndrome may be a predisposing factor for the development of functional tic-like behaviors (functional overlay). Although reports of individual patients presenting with both neurodevelopmental tics and functional tic-like behaviors were rare before the COVID-19 pandemic [85,86], a few studies published in recent years have provided a more accurate characterization of this complex phenotype [35,87,88]. Further research on this subgroup of patients with a dual diagnosis would be useful, based on the updated prevalence figures.

The etiology of functional tic-like behaviors is likely to be multi-factorial; however, it is far from being fully elucidated. As both the psychological burden of the pandemic and increased social media usage have been implicated in the unprecedented rise of functional tic-like behaviors [89], it is advocated that further research is conducted to shed light on the possible role played by exposure to particular social media content [90,91]. Direct social contagion resulting in mass sociogenic illness characterized by tic-like behaviors was documented over a decade ago in adolescents attending a New York State high school [92,93,94]. New technologies and resulting changes in lifestyles and social interactions have led to the re-conceptualization of mass sociogenic illness. In addition to “Mass Social Media Induced Illness” presenting with functional tic-like behaviors [45], an overarching construct, “Social Media Associated Abnormal Illness Behavior” has recently been proposed to encompass different clinical phenotypes facilitated by social media networks [95]. The term “TikTok Tics” has been increasingly used across both clinical encounters and scientific papers [43]. As social media consumption continues to rise, it will be imperative for clinicians to educate patients on the sources and dissemination of medical information [96,97]. This will have practical implications for achieving optimal diagnoses, treatments, and outcomes for patients.

Research in the form of outcome studies with different interventions is needed to determine optimal management for patients with functional tic-like behaviors. Finally, there is a need for further epidemiological studies focusing on the incidence and prevalence of functional tic-like behaviors in the post-pandemic era. It has been suggested that acute presentations of functional tic-like behaviors might have peaked in frequency during the COVID-19 pandemic, based on clinical observations [49] and the Centers for Disease Control and Prevention’s national data from emergency departments [98]; however, monitoring in the post-pandemic phase is recommended. Outbursts in atypical presentations of clinical symptoms resembling neurodevelopmental tics are not new and will likely recur. Therefore, it is important that research efforts are intensified to characterize the clinical phenotypes, clarify the etiological factors, elucidate the underlying mechanisms, trace the long-term outcome, and develop effective treatment interventions.

## 9. Conclusions

The unprecedented rise in adolescents and young adults presenting with acute-onset functional tic-like behaviors during the COVID-19 pandemic has been the focus of recent scientific inquiry. An association with female gender was documented in most studies, especially in countries with increased exposure to female social media influencers portraying similar patterns of functional tic-like behaviors. The available evidence provides useful clinical pointers for the differential diagnosis between functional tic-like behaviors and neurodevelopmental tics as part of Tourette syndrome. These phenomenological features have been incorporated alongside other clinical and demographic factors into two sets of diagnostic criteria. As we transition into the post-pandemic era, several open questions remain. Little is known about the efficacy of treatment interventions beyond psychoeducation. The long-term course of functional tic-like behaviors is the focus of follow-up studies conducted in specialist centers. It is important that newly acquired expertise translates into clinical research to increase awareness about this novel manifestation of a functional neurological disorder and its possible etiological mechanisms.

## Figures and Tables

**Table 1:** Summary of published case series of new-onset functional tic-like behaviors during the COVID-19 pandemic.

Case Series	Year	Country	Setting	Sample Size (F, %)	Age of Onset (Mean, Range)	Main Findings
Hull and Parnes [22]	2021	United States	Specialist clinic	6 (6, 100%)	14 (13–16)	Explosive onset of features incongruous with TS; all patients reported exposure to a specific social media personality before symptom onset
Buts et al. [24]	2022	United Kingdom	Specialist clinic	34 (32, 94%)	14 (8–17)	Co-morbid psychiatric and neurodevelopmental disorders were reported in 91% of patients (anxiety in 68%); previous diagnosis of tics in nearly half of the patients
Fremer et al. [27]	2022	Germany	Specialist clinic	32 (16, 50%)	19 (10–53)	Previous psychiatric symptoms in nearly all patients; 47% of the patients had co-morbid TS
Nagy et al. [28]	2022	Hungary	Specialist clinic	5 (5, 100%)	14 (10–16)	All the patients reported exposure to tic-like behavior on social media before symptom onset
Owen et al. [25]	2022	United Kingdom	Specialist clinic	10 (10, 100%)	12 (9–14)	All the patients had co-morbid psychiatric conditions (mainly anxiety)
Cavanna et al. [26]	2023	United Kingdom	Specialist clinic	105 (76, 72%)	23 (13–63)	Acute/subacute onset in most cases; most common psychiatric co-morbidities: anxiety (70%) and affective disorders (40%); half of the patients reported exposure to tic-like behavior on social media before symptom onset
Firestone et al. [23]	2023	United States	High school	8 (8, 100%)	16 (15–17)	All patients had either prior or ongoing depression or anxiety; abrupt onset in all cases
Martino et al. [30]	2023	International registry	Specialist clinics	294 (255, 87%)	15 (8–53)	Abrupt onset of symptoms in 70% of patients; most common psychiatric co-morbidities: anxiety (66%), depression (28%), ASD (24%), ADHD (23%); over half of the patients reported exposure to tic-like behavior on social media before symptom onset
Okkels et al. [29]	2023	Denmark	Specialist clinic	28 (27, 96%)	14 (11–18)	Most of the patients reported previous trauma/precipitating event (pandemic-related lockdown in 40% of cases); almost two thirds of patients reported psychiatric symptoms/diagnoses; 96% reported exposure to tic-like behavior on social media before symptom onset

Abbreviations. ADHD, attention-deficit and hyperactivity disorder; ASD, autism spectrum disorder; F, female gender; NA, not available; TS, Tourette syndrome.

**Table 2:** Summary of published studies of new-onset functional tic-like behaviors during the COVID-19 pandemic, compared to neurodevelopmental tics.

Clinical Study	Year	Country	Setting	Sample Size—FTLB (F, %)	Age of Onset (Mean, Range)	Comparison Group—NT (F, %)	Main Findings
Paulus et al. [31]	2021	Germany	Specialist clinic	13 (5, 38%)	15 (12–18)	13 (matched)	Compared to patients with NT, patients with FTLB developed after exposure to tic-like behavior on social media reported a more abrupt symptom onset, fewer spontaneous symptom fluctuations, more severe symptoms in the presence of others, more complex movements involving trunk/extremities, later symptom onset
Pringsheim et al. [32]	2021	Canada	Specialist clinic	20 (19, 95%)	14 (13–15)	270 (58, 21%)	Compared to patients with NT, patients with FTLB reported a more abrupt symptom onset, were more likely to be females, older at first visit, older at symptom onset, had higher severity and impairment scores, were more likely to have co-morbid anxiety or depressive disorders; all the patients with FTLB reported exposure to tic-like behavior on social media before symptom onset
Pringsheim and Martino [33]	2021	Canada	Specialist clinic	9 (9, 100%)	15 (11–20)	24 (6, 25%)	Compared to patients with NT, patients with FTLB after exposure to tic-like behavior on social media had an older age at onset, were more likely to be females, reported higher motor and vocal tic severity, more complex arm/hand motor tics, more complex vocal tics (including coprolalia), higher scores on all self-report measures of psychiatric symptoms
Han et al. [34]	2022	Australia	Specialist clinic	22 (22, 100%)	14 (NA)	163 (46, 28%)	Compared to patients with NT, patients with FTLB were more likely to be females, reported more complex tics, later age of onset, higher rates of anxiety/depression; 18% of the patients with FTLB reported exposure to tic-like behavior on social media before symptom onset
Trau et al. [35]	2022	United States	Specialist clinic	36 (34, 94%)	14 (NA)	119 (41, 34%)	A third group of 25 patients had both NT and FTLB (functional overlay); compared to patients with NT, patients with FTLB were more likely to be females, reported more complex and severe tics, more psychiatric co-morbidities; age at tic onset was not significantly different
Andersen et al. [36]	2023	Denmark	Specialist clinic	53 (50, 94%)	14 (6–20)	200 (61, 31%)	Compared to patients with NT, patients with FTLB were more likely to be females, were older at symptom onset, reported more complex tics, were less likely to have family members with tics, were more likely to have family members with a psychiatric disorder, were more likely to have experienced an adverse psychosocial event prior to symptom onset
Baizabal-Carvallo et al. [37]	2023	United States	Specialist clinic	21 (10, 48%)	32 (NA)	156 (34, 22%)	Compared to patients with NT, patients with FTLB were more likely to be females, reported more complex and severe tics, were more likely to be older at symptom onset, were less likely to report motor tics predominantly affecting the head/neck area
Cavanna et al. [38]	2023	United Kingdom	Specialist clinic	83 (59, 71%)	21 (11–61)	83 (matched)	Compared to patients with NT, patients with FTLB were more likely to present their symptoms acutely/subacutely at a later age; reported higher rates of anxiety and other functional neurological disorders; were less likely to report tic-related OCD and a family history of tics
Berg et al. [39]	2024	Canada	Specialist clinic	35 (32, 91%)	17 (NA)	22 (20, 91%)	Patients with FTLB reported significantly higher rates of major depressive disorder and panic disorder and rated their mental health as more severely declined than both patients with NT and neurotypical individuals; overall distress tolerance, resilient coping, suggestibility, hours on social media, and exposure to tic-like behavior on social media were not significantly different between groups
Fremer et al. [40]	2024	Germany	Specialist clinic	32 (16, 50%)	19 (10–53)	1032 (303, 29%)	Compared to patients with NT, patients with FTLB were more likely to be females and older at symptom onset, reported a higher rate of socially inappropriate symptoms, a lower rate of co-morbid ADHD and OCD; rates of co-morbid anxiety and depression and suppressibility of symptoms did not differ between the two groups
Nilles et al. [41]	2024	Canada	Specialist clinic	41 (40, 98%)	16 (11–20)	195 (49, 25%)	Compared to patients with NT, patients with FTLB were more likely to report copropraxia, complex vocal tics (including coprolalia), popping, whistling, simple head movements, self-injurious behaviors
Szejko et al. [42]	2024	Canada	Specialist clinic	40 (31, 78%)	17 (NA)	83 (30, 36%)	Compared to patients with NT, patients with FTLB were more likely to be females, reported more severe tics, were more likely to be older at symptom onset; among patients with FTLB there was a higher prevalence of gender diverse individuals; there were no significant differences in self-reported premonitory urges

Abbreviations. ADHD, attention-deficit and hyperactivity disorder; F, female gender; FTLB, functional tic-like behaviors; NA, not available; NT, neurodevelopmental tics; OCD, obsessive-compulsive disorder.

## Data Availability

No new data were created or analyzed in this study. Data sharing is not applicable to this article.
